# Can social media be beneficial for eating disorders?

**DOI:** 10.1192/j.eurpsy.2021.1861

**Published:** 2021-08-13

**Authors:** G. Lladó Jordan, M.D.C. Díaz García, M. Miguel Cano, M. Jiménez Cubo, B. Lozano Díez, A. Santos Martín, P. Sánchez Esteban, P. Mediavilla Sánchez, J.A. Gómez Del Barrio, R. Ayesa-Arriola

**Affiliations:** 1 Idival, Valdecilla Biomedical Research Institute, Santander, Spain; 2 Uemc, Miguel de Cervantes European University, Valladolid, Spain

**Keywords:** social networks, Pro-Ana, Pro-Mia, eating disorders

## Abstract

**Introduction:**

Eating Disorders are a frequent pathology, particularly among teenagers, a group characterized by its vulnerability and body dissatisfaction. Social networks (SN) can be a gateway to ED, mainly with Pro-Ana and Pro-Mia resources. Despite the aforementioned, SN can also be helpful for professionals, either as a tool of approach to vulnerable groups or as a way of interaction in patients already diagnosed.

**Objectives:**

To study the relationship between ED and SN, using the open access evidence available in Pubmed over the last 5 years.

**Methods:**

A single-phase computerised search was carried out in Pubmed. The search terms were: (“Anorexia Nervosa”[Mesh] OR “Bulimia Nervosa”[Mesh] OR “Feeding and Eating Disorders”[Mesh] OR “Eating Disorders”[Tiab] OR “Eating Disorder”[Tiab] OR “Disorder, Eating”[Tiab] OR “Disorders, Eating”[Tiab] OR “Anorexia”[Tiab] OR “Bulimia”[Tiab]) AND (“blogging”[Mesh] OR “social media”[Mesh]). The filters applied were: “free Full Text” and publications for the last 5 years.

**Results:**

36.84% studied SN as a positive tool for ED. 47.37% revealed negative influence, only 44.44% focused on Pro-Ana and Pro-Mia. 15.79% provided both positive and negative arguments. The most studied SN were Twitter and Facebook.

**Conclusions:**

Despite the known negative effect that SN can have on ED, they can also be used as a supportive recovery framework. They can be used to identify dangerous behaviours and intervene or as a prevention tool.

**Disclosure:**

No significant relationships.

